# The choroid plexus water density

**DOI:** 10.1002/mrm.70028

**Published:** 2025-08-11

**Authors:** Abigail R. Dubois, Maeve Curtin, Kilian Hett, Melanie Leguizamon, Alexander K. Song, Maria Garza, Colin D. McKnight, Ciaran M. Considine, Manus J. Donahue

**Affiliations:** ^1^ Department of Neurology Vanderbilt University Medical Center Nashville Tennessee USA; ^2^ Department of Radiology and Radiological Sciences Vanderbilt University Medical Center Nashville Tennessee USA; ^3^ Department of Psychiatry and Behavioral Sciences Vanderbilt University Medical Center Nashville Tennessee USA

**Keywords:** cerebrospinal fluid, choroid plexus, proton density, water density

## Abstract

**Purpose:**

To quantify normative ranges and circadian variability of the choroid plexus (ChP) water density in healthy adults.

**Methods:**

Actigraphy assessments of circadian activity were performed for 5 days in healthy participants (*n* = 15; age = 28.5 ± 6.5 years) and subsequently participants underwent repeated, high spatial resolution proton density‐weighted imaging (spatial resolution = 0.25 × 0.25 × 1.50 mm) with a driven equilibrium (DRIVE) module at 3 T across four time epochs during wakefulness: 7:00 to 9:00, 11:00 to 13:00, 16:00 to 18:00, and 19:00 to 21:00. ChP water density (unitless ratio of mL water/mL ChP) was calculated as the product of white matter water density and the ratio of the ChP and white matter signal intensity at the level of the atria of the lateral ventricles. Descriptive statistics (mean ± SD; range; median) of water density values at each time were recorded. Spearman and Kendall rank coefficients were used to assess relationships between time, circadian variability, and ChP water density (significance criterion: *p* < 0.05).

**Results:**

Across all participants and scans (*n* = 60), mean ChP water density was 0.895 ± 0.047 (range = 0.806–0.983; median = 0.892). Across time periods, water density was 0.891 ± 0.038 (time = 7:44), 0.891 ± 0.050 (time = 12:17), 0.896 ± 0.045 (time = 16:03), and 0.901 ± 0.058 (time = 19:31), and no relationships between ChP water density and time of day or circadian activity were observed.

**Conclusions:**

The ChP water density at the level of the atria of the lateral ventricles is approximately 0.895 ± 0.047 in healthy adults and does not change significantly with time of day during wakefulness. This value should provide a useful reference for the growing number of neuroimaging protocols that aim to derive quantitative contrast and functional metrics from ChP MRI.

## 1 INTRODUCTION

Choroid plexus (ChP) complexes, located in all four brain ventricles, are composed of choroidal epithelial cells and connecting tight junctions, building an active interface for cellular processes and serving as the blood‐CSF barrier.^1^ The ChP secretes CSF as arterial blood is filtered through the ChP epithelium, producing approximately 430 to 530 mL CSF per day.^2^ Over the past decade, the ChP has gained much attention as the most proximal component of the neurofluid circuit, which has been suggested to have central relevance to the clearance of cerebral peptides and pathological retention of peptides in the setting of neurodegenerative proteinopathies such as Alzheimer, Huntington, and Parkinson diseases.^3^, ^4^ As such, assessing both the structure and function of the ChP is of growing interest in the MRI community, and recent studies have shown abilities to use MRI and deep learning to quantify ChP morphology and hypertrophy in the setting of age and neurodegeneration.^5^, ^6^ Additionally, prior works have used arterial spin labeling (ASL) MRI to demonstrate that ChP perfusion relates to CSF production activity, declines with increasing age, and responds to cortical ischemia.^7^, ^8^, ^9^, ^10^

In terms of ChP function specifically, the ChP is perfused by the anterior and posterior choroidal arteries, which comprise distal branches of vessels that are labeled routinely with both pulsed and pseudo‐continuous ASL (pCASL) methodologies.^11^ Quantification of ChP perfusion (mL blood/100 g tissue/min) from ASL techniques in mice and humans generally proceed by applying kinetic models similar to those applied to gray matter^7^; however, it is well‐known that water exchange and water density will vary between these structures, and as such, additional calibration studies are needed to improve ChP perfusion quantification.^12^, ^13^ ChP water density, a fundamental parameter in the quantification of perfusion from ASL and multiple other MRI sequences, has not been assessed rigorously, and it is generally assumed, but not confirmed, that ChP water density is similar to gray matter water density (˜0.89 mL water/mL gray matter) and blood (˜0.87 mL water/mL blood) water density.^7^, ^9^ Furthermore, similar to the pineal gland, ChP cells are recognized as circadian oscillators.^14^ Considering the filtration nature of the ChP and recent studies indicating diurnal variations in CSF clearance, it is plausible that the water density of the ChP also fluctuates diurnally.^14^, ^15^ Determining whether ChP water density remains stable, or varies throughout the day, will enhance our understanding of how the ChP functions over the circadian cycle of wakefulness. Additionally, repeated assessments of ChP water density will serve as a necessary prerequisite for improving the quantitative accuracy for the growing number of ChP quantitative imaging studies.

The goal of this work is to apply repeated, high spatial resolution proton density‐weighted MRI in healthy adults to quantify ChP water density in the atria of the lateral ventricles over the circadian cycle of wakefulness in healthy adults, thereby providing an important reference for the growing number of functional ChP MRI protocols.

## 2 METHODS

### 2.1 Study design

Healthy, adult participants provided informed consent for this prospective institutional review board (IRB)‐approved study. Before enrollment, participants were screened for undiagnosed sleep disorder symptoms and use of prescription sleep aids, with exclusion criteria including prescription of benzodiazepines, cholinesterase inhibitors, antipsychotics, opioids, or other medications prescribed to help with sleep or sleep disorders, such as insomnia, sleep apnea, narcolepsy, hyper insomnia, circadian rhythm sleep disorder, rapid eye movement behavioral disorder, parasomnia, and sleep‐related movement disorders. Each participant was fitted with a Fitbit (Fitbit Charge 6 Tracker) for at least 5 days before neuroimaging to monitor sleep behaviors and energy expenditure along the 24‐h circadian rhythm. Energy expenditure was measured in the form of metabolic equivalents (METs) from the Fitbit database based on velocity and demographics via a proprietary algorithm. Subsequently, participants were scanned awake four times in the same day during four different time epochs: 7:00 to 9:00, 11:00 to 13:00, 16:00 to 18:00, and 19:00 to 21:00, with a subgroup participating in a repeatability sub‐study where scans were repeated in the same session (within 10 min) after repositioning to understand experimental variation. Participants were instructed to engage in normal activity and sleep habits and not to consume any caffeinated beverages within 12‐h before imaging.

### 2.2 Theory

The signal from a spin echo experiment (*S*) is dependent on the spin density, TR and TE and can be written as

(1) $$S_{i}\left(\mathrm{TR},\mathrm{TE}\right)=A\cdot C_{i}\cdot \left(1-e^{-\mathrm{TR}/T_{1,i}}\right)\cdot e^{-\mathrm{TE}/T_{2,i}},$$

where *i* = the tissue type (i.e., white matter or choroid plexus).

Here, *A* denotes scan‐specific scaling related to signal gain and *C* denotes the water density, which is approximately equivalent to the spin density (often denoted *ρ*_0_) given that the majority of hydrogen nuclei that can be excited are within water molecules. The T_1_ and T_2_ are the longitudinal and transverse relaxation times of the water within the tissue type, respectively. To reduce contributions from T_1_ and T_2_, the proton density experiment is chosen to select TE < < T_2_ and TR > > T_1_. To eliminate sensitivity to the arbitrary scanner calibration factor, *A*, and reduce sensitivity to residual magnetization, the ratio of signal in ChP to white matter can be calculated,

(2) $$\frac{S_{\text{choroid plexus}}}{S_{\text{white matter}}}=\frac{C_{\text{choroid plexus}}}{C_{\text{white matter}}}.$$

Note that white matter is selected here, rather than CSF, given that 3 T white matter T_1_ (approximately 800–1000 ms) and T_2_ (approximately 60–80 ms) values are much shorter than 3 T CSF T_1_ (approximately 4000–4500 ms) and T_2_ (approximately 1000–2500 ms) values and residual T_1_ and T_2_ weighting will remain for species with long relaxation times, such as CSF.^16^, ^17^ Given the high spatial resolution desired for ChP imaging, with long TR and short TE desired, heavily segmented readouts are required and impractical on the timescale of most MRI experiments. To reduce these concerns, a driven equilibrium (DRIVE) module (see Section 2.3) can be applied after the readout to further ensure that longitudinal magnetization is near equilibrium at the time of subsequent excitation. We performed simulations to evaluate how the range of possible white matter water density values and residual magnetizations influenced the ChP water density calculations.

### 2.3 Experiment

**FIGURE 1:**
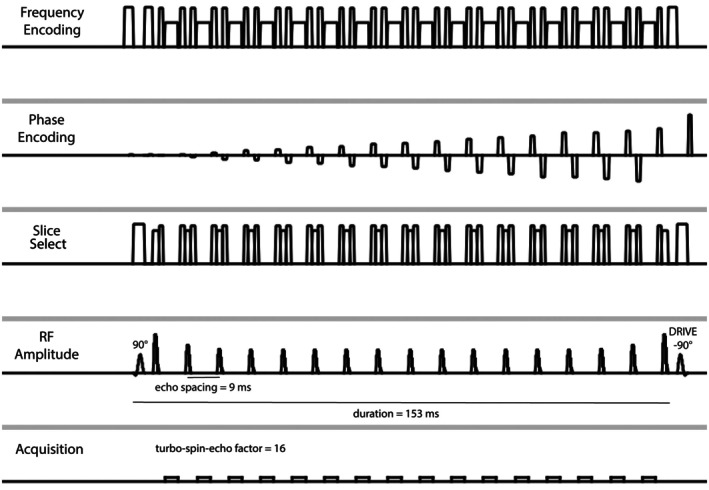
A sequence diagram providing a visual demonstration of the implementation of the segmented turbo‐spin‐echo readout with the concluding driven equilibrium (DRIVE) module. A concluding −90° pulse is applied to restore longitudinal magnetization to near equilibrium. The acquired spatial resolution is 0.25 × 0.25 × 1.5 mm^3^, TE = 9 ms, refocusing angle = 100°, and readout duration per TR = 153 ms. An intermediate TR = 5000 ms is used with the DRIVE module to keep equilibrium choroid plexus and white matter magnetization near equilibrium in a time‐efficient manner (see Section 2 for additional sequence details).

All participants were scanned at 3 T (Elition, Philips Healthcare) using body coil radiofrequency transmission and 32‐channel SENSE phased‐array reception. A non‐contrasted 3D T_2_‐weighted volumetric isotropic turbo‐spin‐echo acquisition (VISTA) sequence with TR = 2500 ms, TE = 331 ms, and spatial resolution = 1 × 1 × 1 mm was acquired for planning of subsequent water density scans. Given that the spatial extent of the ChP is generally 1.5 to 5 mm per direction, and proton density scans require long TRs by the nature of the contrast, in proton density scans we implemented DRIVE modules to ensure that the longitudinal magnetization was near equilibrium for repeated excitations. Specifically, a DRIVE module consisting of a −90° and intermediate TR = 5000 ms to restore the magnetization to equilibrium at the conclusion of the segmented turbo‐spin‐echo readout (factor = 16) was applied. This approach was verified in preliminary studies by comparing the contrast to a long TR = 20 000 ms scan in the absence of the DRIVE module.^18^ Subsequent proton density‐weighted images were acquired with TR = 5000 ms, DRIVE, SENSE = 1.5, TE = 9 ms, spatial resolution = 0.25 × 0.25 × 1.50 mm, echo spacing = 9 ms, turbo‐spin‐echo length per TR = 153 ms, receiver bandwidth = 272.3 Hz, FOV = 180 × 240 mm, slices = 20, slice gap = 0.3 mm, one signal average, and scan duration of 2 min 50 s (Figure 1).

### 2.4 Analysis

For actigraphy, the energy expenditure, measured in METs, was available every minute for a period of 5 to 7 days before imaging. The circadian oscillations from each participant were estimated using a cosinor‐based rhythmometry analysis, with the model defined as

(3) E=α(1+cos(πtf+ϕ)),

where *E* is the energy expenditure, α is the amplitude related to daily energy expenditure, t the time of day expressed in minutes such as t∈[0,1440], f represents the circadian period duration (here, fixed to $\frac{1}{720}$), and ϕ represents the acrophase constraint such as ϕ∈[0,π].^19^ For each participant, the circadian energy model was fit to the measured energy expenditure using non‐linear least squares with the trust region reflective optimization algorithm.^19^ Finally, time of day for each scan expressed in the universal time referential were corrected to each individual circadian time referential to consider individual‐level circadian period.

For ChP water density calculation, manual regions‐of‐interest (ROI), guided by neuroradiology oversight (C.D.M.; experience = 9 years), were used to identify ChP tissue with minimal contribution of nearby CSF, as well as nearby white matter. ITK‐snap was used to perform manual segmentations.^20^ Given the small size of the ChP, a size four, round segmentation tool was used and ChP tissue was segmented within the lateral ventricles, avoiding CSF and vasculature. To reduce sensitivity to spatial variations in shimming and coil sensitivity across the different ROIs, white matter ROIs were placed in periventricular white matter approximately 10 mm from the ependyma of the lateral ventricles. Consistent volumes were used for ChP and white matter ROIs, however, in supplementary analysis the dependency of the calculated ChP water density on the size of the white matter ROI was investigated. Mean signal intensities (SI) for both tissue types were recorded across voxels in each segmentation and were used in the water density calculation. For each time point, ChP water density was calculated as the product of white matter water density (0.70 mL water/mL white matter) and the ratio of the signal intensity in ChP to that in white matter.^21^ White matter was used as the reference region, rather than CSF, given the long T_1_ of CSF (4000–4500 ms) relative to white matter (800–1000 ms) at 3 T and to provide added assurance that longitudinal magnetization fully recovered following the DRIVE pulse.

Finally, to understand how calculated ChP water density values affect the quantification of ChP perfusion from ASL and in the context of prior assumptions, we applied the simplified solution to the flow‐modified Bloch equation for pCASL preparation,

(4) $$f=6000\;\left(\frac{\Delta M}{M_{0,a}}\right)\frac{1}{2\alpha \left(T1_{a}\right)\left(1-e^{-\tau /T1_{a}}\right)e^{-w/T1_{a}}},$$

whereby Δ*M* is the pCASL signal difference magnetization, α=0.85 is the pCASL inversion efficiency, *f* is tissue perfusion (mL/100 g/min), *T1*_α_ is the arterial *T1* of blood = 1.9 s, τ=1.8 s is the labeling duration, and *w* = 2 s is the post‐labeling delay. A factor of 6000 is applied for the conversion of units from mL/g/s to mL/100 g/min. The equilibrium arterial magnetization (*M*_0,a_) is given by the following relationship,

(5) $$\frac{\rho_{\mathrm{ChP}}}{\rho_{a}}=\frac{M_{0,\mathrm{ChP}}}{M_{0,a}},$$

where ρ_ChP_ is water density of the ChP, *ρ*_*α*_ is the water density of arterial blood, and *M*_0,ChP_ is the equilibrium magnetization of the ChP. The common assumption is that arterial and tissue (i.e., ChP, here) equilibrium magnetizations are similar and as such the tissue magnetization is generally used for *M*_0,a_. However, here, we evaluated how the quantified ChP perfusion would change based on the measured ChP water density found in this study, as well as over a range of values from 0.8 to 1.0.

### 2.5 Statistical analysis

Descriptive statistics, including mean, median, and ranges of the signal intensities and quantified ChP water density values were recorded at each time point. A mixed‐effect linear model was used where scan times were used as a group. ChP water density was used as the dependent variable, time related measures as the independent variable, and participants were used as random intercepts. Contrast was assessed for each pair of mean scan times (i.e., 7:44 and 12:17, 12:17 and 16:03, etc.) and a t‐test was used to assess significant differences between mean ChP water density values at each scan time. Spearman's rank and Kendall's τ rank assessments were used to evaluate relationships between scan times and ChP water density values (significance criterion: two‐sided *p* < 0.05).

## 3 RESULTS

For the time‐of‐day assessments, 60 proton density‐weighted scans were performed, divided across 15 participants (age = 28.5 ± 6.5, range = 22–42 years) with four repeated scans each over the circadian cycle of wakefulness. Table S1 in Supporting Information provides a summary of demographic and sleep behavior characteristics. Scans were acquired within the time epochs of 7:00 to 9:00, 11:00 to 13:00, 16:00 to 18:00, and 19:00 to 21:00, with mean scan times of 7:44, 12:17, 16:03, and 19:31. Additionally, five participants (age = 30.0 ± 8.0, range = 22–42 years) underwent two repeated reproducibility measurements to identify normal variability between scans not because of variation with time of day. Average time asleep was 401.07 ± 65.08 min with an average time in bed of 457.88 ± 70.62 min, yielding a mean sleep efficiency of 0.87 ± 0.02.

**FIGURE 2:**
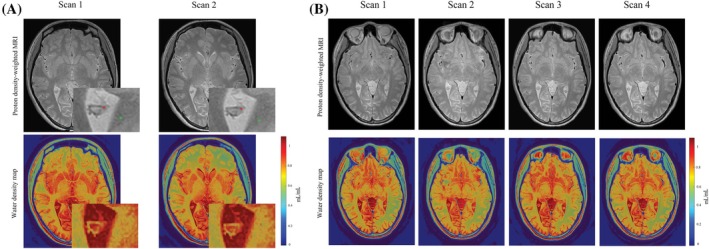
(A) Reproducibility assessments. Two representative proton density‐weighted MRIs with respective quantitative water density maps from one healthy control in the intrasession repeatability study before and after repositioning. Choroid plexus (red) and white matter (green) regions‐of‐interest are shown in the proton density‐weighted scans. Magnifications at the level of the atria of the lateral ventricles are shown as an insert. Water density is shown in units of mL of water/mL of tissue. (B) Circadian assessments. Four representative proton density‐weighted MRIs, from one healthy control, with respective quantitative water density maps at four time points along the circadian cycle of wakefulness. Each scan (1–4) was performed at approximately 7:44, 12:17, 16:03, and 19:31. The color scale is in units of mL of water/mL of tissue. Quantitative values are summarized in Figure 3.

**FIGURE 3:**
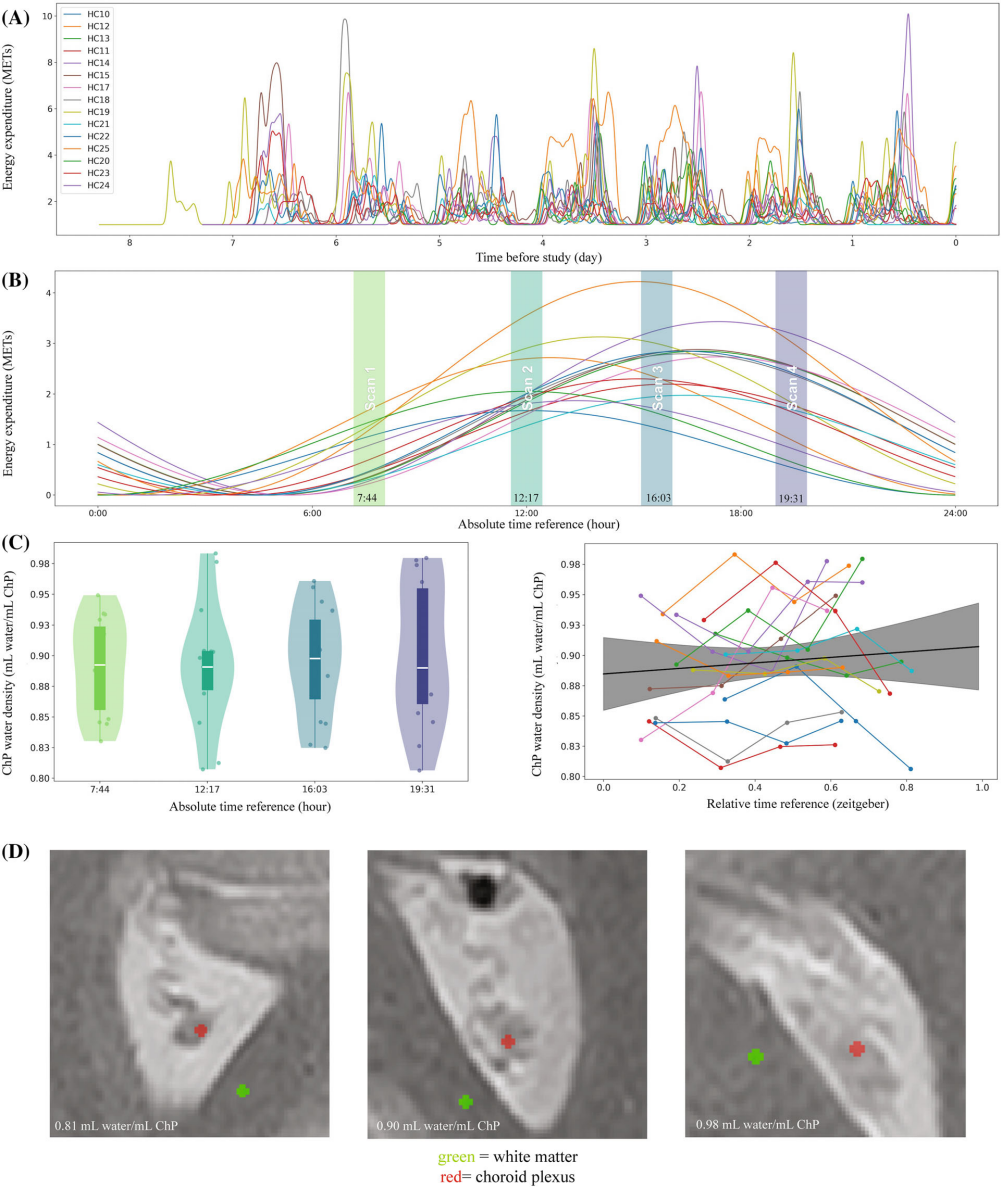
Repeated choroid plexus water density assessments. (A) Energy expenditure, recorded by the Fitbit Charge 6 actigraphy device, is shown as a measure of metabolic equivalents (METs) over multiple days before imaging. Peaks generally denote periods of wakefulness, whereas nadirs largely represent periods of sleep. (B) Circadian energy expenditures for all 15 participants as estimated using a cosinor‐based rhythmometry analysis as it relates to energy expenditure, circadian frequency, and intra‐individual circadian delay. The four scan times are shown along the 24‐h circadian rhythm. This analysis allows for each participant's circadian energy expenditure to be estimated and to provide a reference for comparison with imaging metrics. (C) Mean (left) and individual (right) choroid plexus water density values are shown for all participants at each scan time. The zeitgeber synchronizes the participant's individual circadian rhythm. No significant change was observed across time of day or the circadian cycle of wakefulness, however, relatively large and reproducible inter‐subject variability is observed. (D) Representative manual regions used for three participants with low, middle, and high quantified values do not demonstrate clear differences in CSF partial volume effects (red = choroid plexus; green = white matter), suggesting that inter‐participant variation may be attributable to other factors.

Figure 1 shows the high spatial resolution water density approach, whereas signal intensities and quantified water densities are shown in Table 1, and water density maps from representative participants are shown in Figure 2. Figure 2A shows representative proton density‐weighted images from the reproducibility scans with magnifications at the level of the atria of the lateral ventricles shown as an insert, segmentations of ChP tissue and white matter, and respective water density maps. Reproducibility scans revealed a mean ChP water density of 0.877 ± 0.043 mL water/mL ChP (range = 0.809–0.943 mL water/mL ChP; median = 0.875 mL water/mL ChP). Figure 2B depicts representative proton density‐weighted images from each scan time with their respective water density maps. Across all 15 participants, mean ChP water density was 0.895 ± 0.047 mL water/mL ChP (range = 0.806–0.983 mL water/mL ChP; median = 0.892 mL water/ mL ChP). Using coordinated universal time, no significant change was observed for ChP water density throughout the day (ρ=0.071 *p*‐value = 0.58). Additionally, we investigated the relationship between individual scan time, expressed relative to each subject's internal time reference, and ChP water density measurements. Subject‐relative times were estimated by adjusting universal time timestamps using the phase derived from a cosinor model fitted to individual energy expenditure data. No conclusive relationships were observed between the adjusted scan times and ChP water density measurements (*p*‐value = 0.390). Both mean and individual ChP water density values at each scan time are shown in Figure 3 and results from different region sizes are summarized in Table S2 and Figure S1 of Supporting Information.

**TABLE 1:** Means and SDs for the signal intensities of the choroid plexus and white matter in proton density‐weighted imaging and calculated choroid plexus water densities, at four approximately evenly distributed scan times throughout the day 7:00–9:00, 11:00–13:00, 16:00–18:00, and 19:00–21:00.

	Time: 7:44	Time: 12:17	Time: 16:03	Time: 19:31
Choroid plexus proton density signal intensity (arbitrary units)				
Mean ± SD	600.84 ± 60.49	602.23 ± 60.41	574.04 ± 61.74	578.78 ± 65.70
Range (min–max)	504.21–724.51	480.16–695.29	450.28–662.79	448.86–711.31
White matter proton density signal intensity (arbitrary units)				
Mean ± SD	473.04 ± 52.15	474.67 ± 59.79	449.61 ± 53.24	450.53 ± 54.62
Range (min–max)	377.99–556.16	372.02–598.96	354.13–527.36	389.64–563.74
Choroid plexus water density (mL water/mL choroid plexus)				
Mean ± SD	0.891 ± 0.038	0.891 ± 0.050	0.896 ± 0.045	0.901 ± 0.058
Range (min–max)	0.830–0.949	0.807–0.983	0.825–0.961	0.806–0.980

*Note*: Mean scan times were 7:44, 12:17, 16:03, and 19:31.

Abbreviations: max, maximum; min, minimum.

When a separate scan with a conventional TR = 20 000 ms without the DRIVE module was considered, it was observed that the signal intensities for white matter and ChP were 2.17% and 2.19% higher, respectively, compared to an intermediate TR = 5000 ms with the DRIVE module. This led to an approximately 0.018% error in calculated water density when comparing the intermediate TR = 5000 ms with DRIVE to the conventional long TR = 20 000 ms sequence. Given the calculated error of 0.018%, these findings are consistent with the time‐efficient DRIVE scan contributing to only a small variation in the calculated mean ChP water density of 0.003 mL water/mL ChP. Additionally, we explored the size of the white matter ROI in a subgroup of participants (see Data S1) and observed no significant difference in the calculated ChP water density between the smaller (volume = 1.24 ± 0.00 mm^3^) and larger (volume = 67.00 ± 9.86 mm^3^) ROI volumes assessed.

**FIGURE 4:**
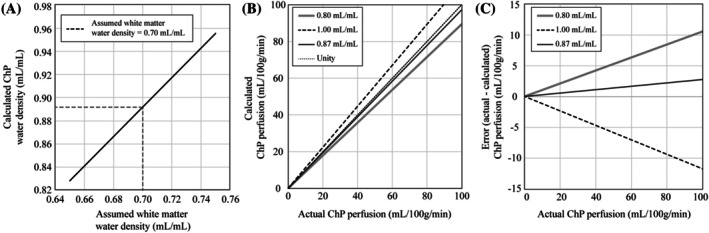
Simulations and considerations for experimental measures. (A) The calculated choroid plexus (ChP) water density depends linearly on the assumed white matter water density in the proposed quantification procedure. This work assumes a mean white matter water density of 0.70 mL/mL (dashed line) based on literature, however, the plot shows how the calculated ChP water density would vary over a reasonable range of assumed white matter water density values from 0.64–0.75 mL/mL. (B) How the calculated ChP perfusion would vary, relative to the actual ChP perfusion using the measured ChP water density of 0.895 mL/mL, here, for the range of ChP water density values from 0.80 to 1.00 mL/mL. Note that we assume a blood water density of 0.87 mL/mL from the literature. (C) There is close similarity in calculated and actual ChP perfusion values when assuming an identical ChP and blood water density (thin gray line), as has been assumed in previous ChP perfusion work, of approximately 0.5–3 mL/100 g/min over a physiological range of 35–75 mL/100 g/min.

Finally, we assumed a literature white matter water density value of 0.70 mL water/mL white matter for calibration. On simulation over a white matter water density range from 0.65 to 0.75 mL water/mL white matter, the calculated ChP water density was 0.833–0.955 mL water/mL ChP (Figure 4A). Figure 4B,C also summarizes how different assumptions for ChP water density affect the calculated perfusion values from a typical pCASL experiment. These simulations demonstrate that over a typical perfusion range of 35 to 70 mL/100 g/min, using the measured ChP versus assuming an identical tissue and arterial blood water density and equilibrium magnetization, leads to an error of approximately 0.5 to 3 mL/100 g/min.

## 4 DISCUSSION

We performed repeated proton density‐weighted scans across the circadian cycle of wakefulness to quantify ChP water density as a function of time of day and circadian energy expenditure in healthy adults. Findings indicate that ChP water density in the atria of the lateral ventricles, where the majority of ChP resides, is approximately 0.895 ± 0.047 mL water/mL ChP and does not change significantly with time of day or circadian energy expenditure, although does vary more widely across individuals. This work provides, to our knowledge, the first repeated assessment of ChP water density in humans.

ChP perfusion imaging has received growing attention, as vessels perfusing the ChP (anterior and posterior choroidal arteries) are distal branches of vessels routinely labeled with ASL MRI. As such, perfusion assessments from ASL have been reported in both mice and humans.^7^, ^9^, ^22^ These studies require application of the flow‐modified Bloch equation to quantify perfusion, which includes a term for the ChP water density as a component of the calibration procedure with arterial blood water. In these and similar studies, it has always been assumed, but not confirmed, that ChP water density is similar to gray matter water density. Here, we demonstrate that this assumption is approximately valid on average, and also that ChP water density does not vary beyond experimental error with time of day. Findings from this study should complement known water densities for gray matter (0.89 mL water/mL gray matter), white matter (0.70 mL water/mL white matter), and CSF (1 mL water/mL CSF) that are frequently used in quantitative imaging.^17^, ^23^ Because the known water density for white matter was used in the ratio approach to determine ChP water density, variations in the water density of white matter across participants would impact calculated ChP water density. We also simulated ChP water density for a reasonable range of white matter water densities for completeness (Figure 4). Future work in ASL of the ChP will likely expand exchange models to include both blood and tissue water exchange, but also blood to CSF filtration. Improving the quantitation of these methods will require accurate values for all equation parameters, and the quantified water density of the ChP should hopefully provide an important value for this approach. Furthermore, the comparatively large inter‐subject variation in ChP water density, which was largely stable across repeated measures, suggests that differences in filtration may exist between individuals and ASL experiments may benefit from including a water density calibration scan.

This work also raises the question as to whether ChP water density is homogeneous or heterogeneous throughout ChP tissue. As the ChP translates blood plasma into CSF, it may be reasonable to assume that ChP water density is not homogeneous, specifically between medial and lateral aspects of the ChP. Therefore, future research should aim to investigate how this potential inhomogeneity of concentration is distributed as ChP volume increases, and how this may affect ChP perfusion calculations, especially in elderly adults and patients with neurodegeneration. Although we used a relatively high spatial resolution here (0.25 × 0.25 × 1.5 mm), such questions will likely require even higher spatial resolution to be addressed rigorously.

We observed no dependence of water density on time of day. To ensure that participants had normal sleep habits, we monitored sleep behaviors before the study, which showed participants exhibited normal sleep behaviors, consistent with prior actigraphy‐based characterization of sleep, in healthy adults.^24^ Because we observed no changes over the time of day, we did not perform detailed water density assessments in relation to sleep behaviors. However, this could be a topic of future work in patients with sleep dysfunction, as well as how ChP water density relates to other metrics of the neurofluid circuit, including ChP perfusion and CSF flow itself. Measures of sleep efficiency should continue to be included in analyses and are important to consider as sleep is a major component of the 24‐h circadian rhythm, and CSF production has been shown to increase during sleep for metabolic waste clearance.^25^

Several limitations of this work should also be noted. First, we used an intermediate TR = 5000 ms protocol, which will allow insufficient time for magnetization in long TR species such as CSF to fully recover. However, we incorporated a DRIVE module and validated this approach against a TR = 20 000 ms sequence and also used a ratio approach including white matter (with short T_1_ and T_2_ relative to the TR and TE used here) to reduce errors. In independent validation studies with this long TR = 20 000 ms sequence, we confirmed that water density measures differed by only 0.018%, thereby motivating future work that seeks to obtain proton density‐weighted scans of the ChP at high spatial resolution with a similar protocol as described here. Second, we only evaluated variability during wakefulness. It is possible that water density may change during sleep, especially given the recently reported upregulation of the neurofluid circuit during sleep.^26^ Finally, measurement error may arise from a shifting of head placement between scans or residual partial volume effects. These confounds were reduced by performing manual segmentations and using high spatial resolution. Additionally, to avoid signal heterogeneity, manual segmentations were performed in the purest ChP tissue identifiable, avoiding contributions from CSF and vasculature. Of the 15 participants, seven had ROIs drawn in the right hemisphere and eight had ROIs drawn in the left hemisphere. Laterality was case dependent and not accounted for in the analysis, although in our prior work of healthy individuals we have found no evidence of a laterality effect in functional or structural measures.

In conclusion, we performed repeated high spatial resolution water density assessments of the ChP in humans, observing that the ChP water density is approximately 0.895 ± 0.047 mL water/mL ChP and does not change beyond measurement error during the circadian cycle of wakefulness. This value should provide a useful reference and sequence for the growing number of quantitative ChP MRI studies.

## CONFLICT OF INTEREST STATEMENT

M.J.D. is a paid consultant for Pfizer, Global Blood Therapeutics (now Pfizer), Woolsey Pharmaceuticals, Graphite Bio, and LymphaTech, and Huntington's Study Group (HSG); recently served on the advisor boards of Pfizer, Novartis, and Bluebird Bio; receives research‐related support from Philips Healthcare and research funding from Pfizer, and the National Institutes on Health (National Cancer Institute, National Institute of Neurological Disorders and Stroke, National Heart, Lung, and Blood Institute, National Institute of Nursing Research, National Institute on Aging, and National Collaborating Centre for Indigenous Health); and is the Chief Executive Officer of Biosight, which operates as an imaging clinical research organization. These agreements have been approved by Vanderbilt University Medical Center in accordance with its conflict‐of‐interest policy.

## FUNDING INFORMATION

National Institute on Aging, Grant/Award Numbers: 5R01AG083159 and 5R01AG062574.

## Supporting information


**Data S1** Supporting Information.
