# Supplementary material for: The choroid plexus water density
Source: Magn Reson Med. 2025 Aug 11;95(1):545–54. doi: 10.1002/mrm.70028 (PMC12510407; doi:10.1002/mrm.70028)
Supplement: Supplementary file 1 — Data S1 Supporting Information. [file MRM-95-545-s001.docx]

**SUPPLEMENTARY INFORMATION**

| **Healthy Participants** |  |
| --- | --- |
| Number | 15 |
| Sex (Male/Female) | 7/8 |
| Race (White/Asian) | 13/2 |
| Age (Years) | 28.53 ± 6.53 |
| **Actigraphy Sleep Measurements** |  |
| Time asleep (minutes) | 401.1 ± 65.1 |
| Time in bed (minutes) | 457.9 ± 70.6 |
| Sleep efficiency (minutes) | 0.87 ± 0.02 |
| Time in light sleep (minutes) | 229.5 ± 80.2 |
| Time in deep sleep (minutes) | 66.0 ± 25.7 |
| Time in REM^1^ (minutes) | 77.3 ± 24.5 |

^1^ REM = rapid-eye-movement sleep

**Table S1. (Top)** Demographic characteristics (means and standard deviations) for all healthy controls in the circadian time-of-day study. **(Bottom)** Sleep measures recorded with Fitbit Charge 6 Trackers. All values are reported as counts for categorical measures or mean +/- standard deviation for continuous measures.

| Participant ID | ROI | Volume of ROI (mm^3^) | Signal intensity | ChP water density  (mL water/mL ChP) |
| --- | --- | --- | --- | --- |
| HC10 | Small WM ROI | 1.24 | 481.68 | 0.84 |
|  | Large WM ROI | 79.45 | 478.68 | 0.84 |
| HC12 | Small WM ROI | 1.24 | 488.02 | 0.86 |
|  | Large WM ROI | 63.38 | 487.48 | 0.86 |
| HC14 | Small WM ROI | 1.24 | 451.49 | 0.91 |
|  | Large WM ROI | 75.43 | 462.33 | 0.89 |
| HC17 | Small WM ROI | 1.24 | 474.17 | 0.88 |
|  | Large WM ROI | 57.81 | 472.17 | 0.88 |
| HC18 | Small WM ROI | 1.24 | 469.96 | 0.85 |
|  | Large WM ROI | 58.94 | 483.76 | 0.83 |

**Table S2.** Variation in recorded white matter signal intensities and calculated choroid plexus (ChP) water density values between the small ROIs (size 4, round) used in the experiment and larger ROIs. Large ROI segmentations were performed to maximize coverage of pure periventricular white matter, and the volume of each segmentation was recorded. Signal intensities were measured in five participants. No significant differences were found between calculated choroid plexus water densities with small (volume=1.24±0.00 mm^3^) and large (volume=67.00±9.86 mm^3^) ROIs (p-value=0.68).


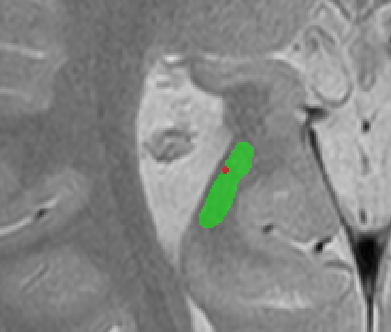


**Figure S1.** A representative example of white matter ROIs are shown for one participant.
